# Protocol for differentiation of vascular smooth muscle cells from human iPSCs and their application in CRISPRa-mediated gene regulation

**DOI:** 10.1016/j.xpro.2025.104345

**Published:** 2026-01-21

**Authors:** Laura Priesmeier, Malte Tiburcy, Laura Cecilia Zelarayán

**Affiliations:** 1 Institute of Pharmacology and Toxicology, University Medical Center Goettingen, 37075 Lower Saxony, Germany; 2 German Center for Cardiovascular Research (DZHK), Lower Saxony, Germany; 3 Clinic for Cardiology and Pneumology, University Medical Center Goettingen, 37075 Lower Saxony, Germany; 4 Justus-Liebig-University Giessen, Medical Clinic I, Department of Cardiology and Angiology, 35392 Hesse, Germany

**Keywords:** Cell culture, Gene expression, CRISPR, Cell differentiation

## Abstract

Directed differentiation of human induced pluripotent stem cells (hiPSCs) holds major promise for the development of disease models, drug screening platforms, and regenerative medicine. Here, we provide a step-by-step highly reproducible protocol for differentiating vascular smooth muscle cells (vSMCs) from hiPSCs, including hiPSC culture, hiPSC differentiation, and vSMC passaging under chemically defined conditions. We also detail molecular and functional analysis procedures for hiPSC-derived contractile vSMCs along with endogenous transcriptional activation modulation ready for any downstream application.

## Highlights

• Steps for generating vSMCs from hiPSCs in chemically defined conditions
• Procedures for differentiating epicardial-like intermediates into vSMCs
• Guidance on assessing contractility using collagen gel contraction assays
• Instructions for molecular characterization and CRISPRa in vSMCs

## Abstract (editor highlights)

Publisher’s note: Undertaking any experimental protocol requires adherence to local institutional guidelines for laboratory safety and ethics.

## Graphical abstract

**Figure:**
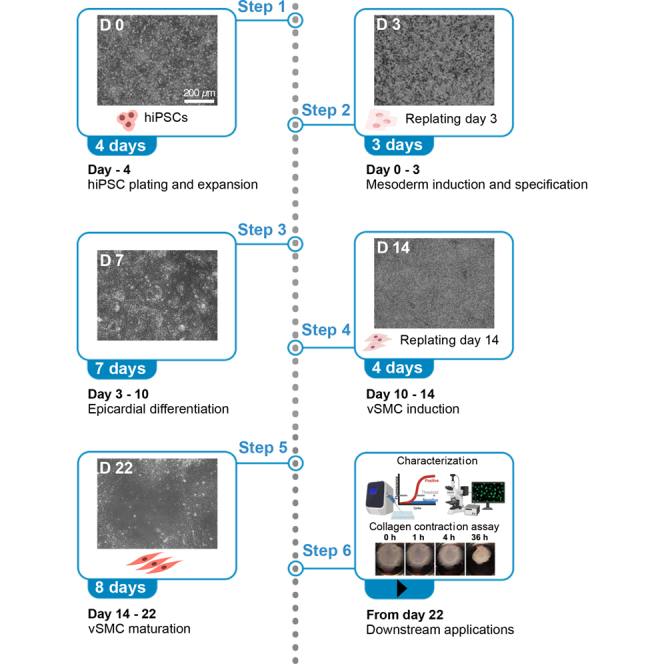

## Before you begin

This protocol was developed and optimized to generate contractile vSMCs from hiPSCs for downstream functional and molecular analyses. In its current form, the protocol has been validated using collagen gel contraction assays and by assessing expression of vSMC markers together with expression changes in markers associated with epicardial development. The technique is compatible with downstream genetic perturbation approaches. As a proof of concept, gene modulation was performed in differentiated vSMCs via CRISPR activation (a) approach.

Minor adaptations to the protocol may be required when applying the protocol to different hiPSC lines.

### Plate coating for hiPSC maintenance and mesoderm differentiation

**Timing: 30 min**

hiPSC maintenance and differentiation towards vSMCs is preferably performed on Matrigel-coated plates.

1. Place a bottle of Growth Factor Reduced Matrigel (approximately 80–100 mg in 10 mL) on ice to thaw. Divide the Matrigel into smaller portions, such as 1 mL (containing about 8–10 mg). Transfer these aliquots into Eppendorf tubes to be stored at −80°C.
2. Use 119 mL ice-cold DPBS (−/−) to dissolve one aliquot (1 mL) of frozen Matrigel and dilute it 1:120 in a pre-chilled 50 mL falcon tube.
3. Immediately dispense the diluted Matrigel into flasks at a volume-to-surface density of 100 μL/cm^2^. Ensure that the Matrigel stock remains on ice throughout the process and avoid using it if it has warmed to 20°C or higher.
4. After distributing the Matrigel, the plates can be used after an incubation period of either 1 h at 20°C or 30 min at 37°C. Cover the plates with foil to prevent drying out and store them at 4°C for up to 1 month. This protocol ensures effective preparation and use of Matrigel for your experimental needs.

### Alternative coating for hiPSC maintenance and coating instructions for epicardial and vSMC derivatives

#### Vitronectin

5. Defrost a 1 mL vial of Recombinant Human Vitronectin (VTN-N) (500 μg/mL) that has been stored at −80°C. Divide the vial into four equal portions of 0.25 mL each for future use.
6. Dilute each 0.25 mL aliquot in 49.75 mL of DPBS (−/−).
7. Use this diluted solution to coat flasks at a volume-to-surface density of 200 μL/cm^2^. Ensure the Vitronectin stock remains on ice throughout the process and avoid using it if it has warmed to 20°C or higher.
8. After Vitronectin distribution, flasks can be used after an incubation period of 30 min at 37°C and 1 h at 20°C. Cover the plates with foil to prevent them from drying out and store at 4°C for up to 1 week. Discard plates where the Vitronectin coating has dried out.

#### Laminin 521

9. Thaw an aliquot of Laminin 521 (LN521) by placing it on ice from −20°C.
10. In a pre-chilled sterile container, dissolve one aliquot (5 mL) of LN521 using 195 mL of ice-cold DPBS (+/+), diluting it to a ratio of 1:40.
11. Distribute the LN521 dilution into flasks at a volume-to-surface density of 120 μL/cm^2^. Keep the LN521 stock on ice at all times, and do not use it if it has warmed to 20°C or higher.
12. After distributing the LN521, allow the flasks to incubate for 1 h at 20°C or for 30 min at 37°C. Cover the plates with foil to prevent them from drying out and store them at 4°C for up to 1 week. Any plates where the LN521 coating has dried out should be discarded.

**CRITICAL:** Since these reagents begin to gel at temperatures above 10°C and solidify quickly at 20°C, it is crucial to keep the materials on ice during the entire coating process.

***Note:*** Matrigel, which is sourced from mouse sarcoma, contains non-human components and is not chemically defined. This lack of definition poses potential challenges for clinical use.^1^ As a result, each batch must be evaluated for its effects on hiPSC cultures before being used extensively. In comparison, VTN-N and LN521 are composed entirely of recombinant human proteins and are chemically defined, making them a more appropriate choice for clinical applications.^2^^,^^3^^,^^4^ Replacing Matrigel with VTN-N or LN521 during hiPSC expansion and the initial days of differentiation renders the entire process chemically defined.

### Innovation

**Figure 1:**
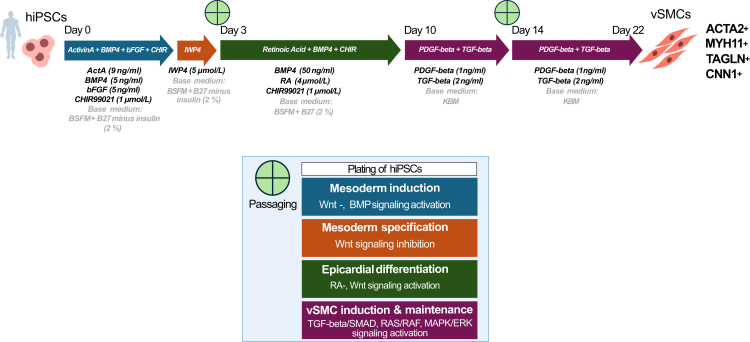
vSMC differentiation strategy for hiPSCs Schematic diagram illustrating the differentiation of vSMCs from hiPSCs through modulation of key signaling pathways, indicated by arrows and colors. The induction of lineage-committed vSMCs is represented with corresponding molecular markers (ACTA2, MYH11, TAGLN and CNN1).

This protocol introduces a fully defined hiPSC-derived vSMC differentiation workflow that integrates functional assessment and genetic perturbation without additional modification steps. A central advancement is the incorporation of collagen gel contraction assays as a standard validation readout, enabling direct assessment of contractile behaviour rather than reliance on molecular markers alone. The differentiation strategy is designed to proceed through epicardial-like intermediates, reflecting key features of developmental vSMC emergence and enabling analysis of lineage progression across defined stages (Figure 1). This developmental framing distinguishes the workflow from endpoint-focused differentiation approaches.

In addition, the protocol demonstrates compatibility of differentiated vSMCs with CRISPRa-based gene activation via lentiviral transduction, indicating suitability for studying gene regulatory mechanisms and vascular phenotypes in a human cellular context.

## Key resources table

**Table:** 

REAGENT or RESOURCE	SOURCE	IDENTIFIER
**Antibodies**		

SM-MHC11 (host: rabbit), dilution 1:100	Abcam	ab125884
TAGLN (host: rabbit), dilution 1:100	Abcam	ab14106
SMA (host: mouse), dilution 1:100	Agilent Dako	GA611
Dylight 550 goat anti-rabbit IgG, dilution 1:100	Abcam	ab96884
Alexa Fluor 488 goat anti-mouse IgG, dilution 1:100	Invitrogen	A-11001
DAPI, dilution: 1:1000	Sigma-Aldrich	D9542

**Chemicals, peptides, and recombinant proteins**		

DPBS (no calcium, no magnesium; −/−)	ThermoFisher	14190144
DPBS (plus calcium, plus magnesium; +/+)	Thermo Fisher	14040083
StemMACS Brew XF, human	Miltenyi Biotec	130-104-368
ROCK inhibitor Y27632	Stemgent	04-0012-10
Versene	ThermoFisher	15040066
Matrigel	Corning	354237
RPMI 1640 + GlutaMax	ThermoFisher	61870036
Pencillin/streptomycin	ThermoFisher	15140122
L-Ascorbic acid	SigmaAldrich	A8960
Sodium Pyruvate	ThermoFisher	11360070
B27	ThermoFisher	17504044
B27 minus insulin	ThermoFisher	A1895601
CHIR99021	Stemgent	04–0004
Activin A	R&D Systems	338-AC
BMP4	R&D Systems	314-BP
bFGF	ThermoFisher (PeproTech)	PHG0264
IWP-4	Stemgent	04–0036
TrypLE Express	ThermoFisher	12604013
Laminin 521	BioLamina	LN521-05
Retinoic Acid	SigmaAldrich	R2625
Vitronectin	Invitrogen	A14700
KnockOut DMEM	ThermoFisher	10829018
KnockOut Serum	ThermoFisher	10828028
L-Glutamine	ThermoFisher	A2916801
PDGF-beta	ThermoFisher (Peprotech)	AF-100-14B
TGF-beta 1	ThermoFisher (Peprotech)	100–21
PFA	Carl ROTH	0335.5
Triton-X	ThermoFisher	11358311
Takyon SYBR Mix	Eurogentech	UF-RSMT-B0701

**Experimental models: Cell lines**		

LiPSC-GR1.1	Lonza	–
TC-1133-CRISPRa2 (RUCDRi002-A-15 (CRISPRa2))	Derived from LiPSC-GR1.1	–
**qPCR primers**		
*OCT4*	*Fwd* CAGTGCCCGAAACCCACAC *Rev* GGAGACCCAGCAGCCTCAAA	–
*NANOG*	*Fwd* CAGAAGGCCTCAGCACCTAC *Rev* ATTGTTCCAGGTCTGGTTGC	–
*TBX18*	*Fwd* GCTCTGGAAGCGCTTTCATG *Rev* GGCCTTGGTCATCCAGTTCA	–
*SNAI1*	*Fwd* TAGCGAGTGGTTCTTCTGCG *Rev* AGGGCTGCTGGAAGGTAAAC	–
*MYH11*	*Fwd* CAGGGTCAAGCAGCTCAAGA *Rev* GCCGTGGTGCAAAACTGTAG	–
*CNN1*	*Fwd* CAGCATGGCGAAGACGAAAG *Rev* CCGCCCTTCTCTTAGCTTCC	–
*TAGLN*	*Fwd* TGGCTGAAGAATGGCGTGAT *Rev* CCACGGTAGTGCCCATCATT	–
*IGFBP5*	*Fwd* TGCACCTGAGATGAGACAGG *Rev* GAATCCTTTGCGGTCACAAT	–
**gRNA oligonucleotide sequences**		
*IGFBP5-*B	*Fwd* GGCGCTGTTCAGGGAGCGAA *Rev* TTCGCTCCCTGAACAGCGCC	–
*IGFBP5-*C	*Fwd* GCGCTGTTCAGAGGGGAGGA *Rev* TCCTCCCCTCTGAACAGCGC	–
*IGFBP5-*D	*Fwd* GCCCCTTTCTTACATTCCGG *Rev* CCGGAATGTAAGAAAGGGGC	–
NT-1	*Fwd* GTCCAGCGGATAGAATGGCG *Rev* CGCCATTCTATCCGCTGGAC	–
NT-2	*Fwd* GGAGCGGTTTTGGATATTAG *Rev* CTAATATCCAAAACCGCTCC	–
NT-3	*Fwd* GTATGAGCGCGATGAAGGTG *Rev* CACCTTCATCGCGCTCATAC	–

**Software and algorithms**		

SDS2.4	Thermo Fisher Scientific	https://www.thermofisher.com/de/de/home/technical-resources/software-downloads/applied-biosystems-7900ht-fast-real-timespcr-system.html
GraphPad Prism	GraphPad, Version 8.4.2	https://www.graphpad.com/

**Other**		

PCR machine	AB7900 HT Fast Real-Time PCR system, Applied Biosciences	–
Centrifuge	Heraeus Megafuge 40R	–
Incubator	Heracell 240i	–

## Materials and equipment

Rock Inhibitor (Y27632) 10mM stock solution: In a sterile environment, dissolve 100 mg of Y27632 in 31.2 mL of DMSO. Store in 200 μL aliquots and avoid freeze-thaw cycles. Keep protected from light.

CHIR99021 10mM stock solution: In a sterile environment, dissolve 5 mg of CHIR99021 in 1075 μL of DMSO. Incubate at 37°C for 5 min. Store in 100 μL aliquots and avoid freeze-thaw cycles.

Basic fibroblast growth factor (bFGF) 10 μg/mL stock solution: In a sterile environment, dissolve 1 mg of bFGF in 1 mL of sterile filtered H_2_O and add 99 mL of 0.1 % HSA (in PBS). Store in 500 μL aliquots and avoid freeze-thaw cycles.

Activin A (ActA) 10 μg/mL stock solution: In a sterile environment, dissolve 1 mg of ActA in 100 mL 4 mM HCl. Store in 500 μL aliquots and avoid freeze-thaw cycles.

Bone morphogenetic protein 4 (BMP4) 10 μg/mL stock solution: In a sterile environment, dissolve 1 mg of BMP4 in 99 mL of 4 mM HCl and add 1 mL of sterile filtered 10 % HSA (in H_2_O). Store in 500 μL aliquots and avoid freeze-thaw cycles.

Inhibitor of Wnt Production-4 (IWP4) 5 mM stock solution: In a sterile environment, dissolve 50 mg of IWP4 in 20.14 mL of DMSO. Incubate at 37°C until IWP4 is completely dissolved. Store in 500 μL aliquots and avoid freeze-thaw cycles.

Retinoic Acid (RA) 8 mM stock solution: In a sterile environment, dissolve 50 mg of RA in 1.67 mL of DMSO (100 mM stock). Dilute further 1:12.5 in 100 % ethanol. Store in 100 μL aliquots and avoid freeze-thaw cycles. Protect from light.

Transforming growth factor beta 1 (TGF-beta 1) 5 μg/mL stock solution: In a sterile environment, dissolve 100 μg of TGF-beta 1 in 0.5 mL of sterile filtered H_2_O and add 19.5 mL of 0.1 % HSA (in PBS). Store in 100 μL aliquots and avoid freeze-thaw cycles.

Platelet-derived growth factor beta (PDGF-beta) 5 μg/mL stock solution: In a sterile environment, dissolve 50 μg of PDGF-beta in 1 mL of sterile filtered H_2_O and add 9 mL of 0.1 % HSA (in PBS). Store in 50 μL aliquots and avoid freeze-thaw cycles.

L-Ascorbic Acid (L-Asc) 200 mM stock solution: In a sterile environment, dissolve 1.16 g of L-Ascorbic acid 2-phosphate sesquimagnesium salt hydrate in 20 mL of sterile filtered H_2_O and vortex thoroughly. Place on a shaker at room temperature for 1 h. Store in 1 mL aliquots and avoid freeze-thaw cycles.

hiPSC culture medium: StemMACS Brew XF, supplemented with 100 U/mL penicillin and streptomycin.

### Media compositions

**Table:** Basal Serum Free Medium (BSFM)

Reagent	Final concentration	Amount
L-Ascorbic Acid	200 μmol/L	510 μL
Sodium pyruvate	1 mmol/L	5,1 mL
Penicillin/streptomycin	100 U/mL	5,1 mL
RPMI 1640 plus GlutaMax	N/A	500 mL
**Total**	**N/A**	**510,71 mL**

Store at 4 °C for up to 4 weeks.

**Table:** Mesoderm Induction Medium (MIM)

Reagent	Final concentration	Amount
B27 supplement minus insulin	2 %	1 mL
CHIR99021	1 μmol/L	5 μL
bFGF	5 ng/mL	25 μL
BMP4	5 ng/mL	25 μL
ActA	9 ng/mL	45 μL
BSFM	N/A	48,9 mL
**Total**	**N/A**	**50 mL**

Prepare fresh.

**Table:** Mesoderm Specification Medium (MSM)

Reagent	Final concentration	Amount
B27 supplement minus insulin	2 %	1 mL
IWP4	5 μmol/L	50 μL
BSFM	N/A	48,95 mL
**Total**	**N/A**	**50 mL**

Prepare fresh.

**Table:** Epicardial Specification Medium (ESM)

Reagent	Final concentration	Amount
B27 supplement	2 %	1 mL
CHIR99021	1 μmol/L	5 μL
BMP4	50 ng/mL	250 μL
RA	4 μmol/L	25 μL
BSFM	N/A	48,72 mL
**Total**	**N/A**	**50 mL**

Prepare fresh.

**Table:** KnockOut Basal Medium (KBM)

Reagent	Final concentration	Amount
KO-SR	10 %	56 mL
L-Glutamine	2 mmol/L	5,6 mL
Pencillin/streptomycin	100 U/mL	5,6 mL
KnockOut DMEM	N/A	500 mL
**Total**	**N/A**	**567,2 mL**

Store at 4°C for up to 4 weeks.

**Table:** Smooth Muscle Medium (SMM)

Reagent	Final concentration	Amount
TGF-beta	2 ng/mL	20 μL
PDGF-beta	1 ng/mL	10 μL
KBM	N/A	49,97 mL
**Total**	**N/A**	**50 mL**

Prepare fresh.

## Step-by-step method details

### Maintenance and passaging of hiPSCs

**Timing: 30 min**

This step ensures hiPSCs are maintained under defined conditions and seeded at appropriate density to enable reproducible and synchronized differentiation.

**Figure 2:**
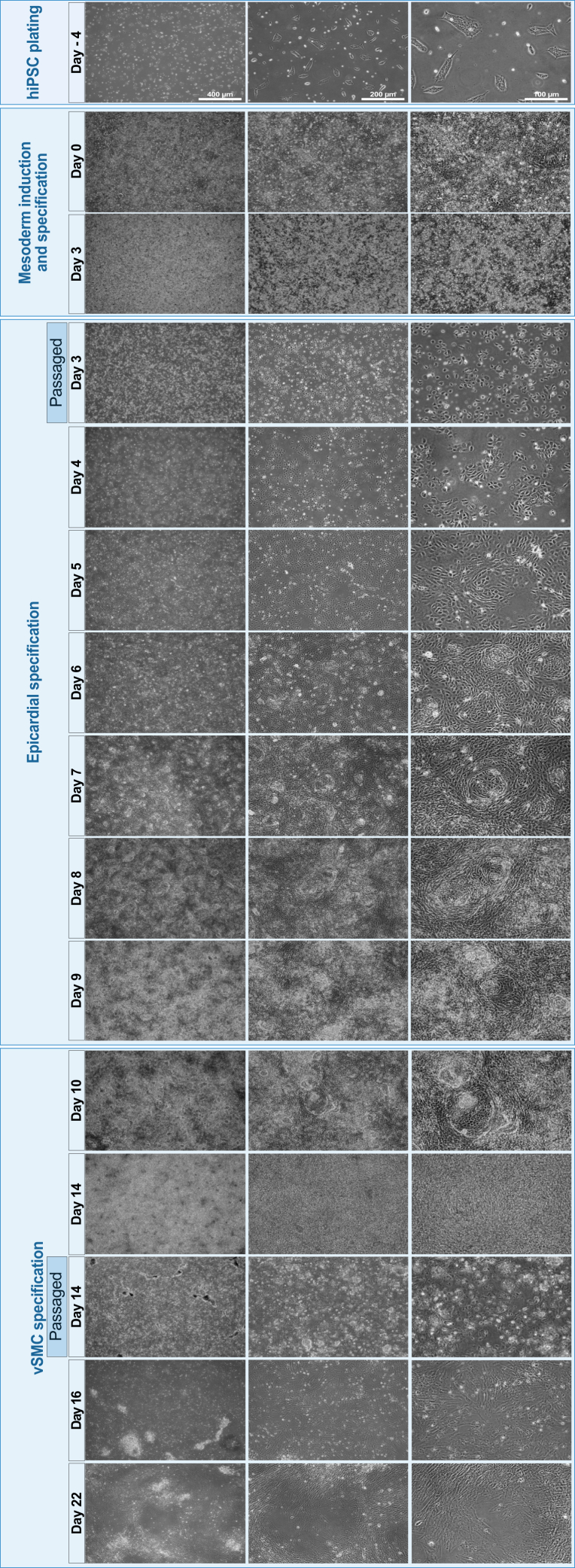
hiPSC-derived vSMC differentiation Phase contrast microscopy showing the chronologically morphological changes of vSMC progenitors throughout differentiation.

Passage hiPSCs twice a week with daily medium changes to maintain a fully pluripotent state over several passages. Check cell-morphology regularly. Cell morphology should resemble the shape shown in Figure 2.

1. To passage hiPSCs at 80 % confluency, aspirate StemMACS Brew medium and rinse the cells with 3 mL of DBPS per T25 flask to remove excessive medium.
2. Aspirate DPBS and add 3 mL of Versene (prewarmed to 37°C) per T25 flask. Leave at 37°C for 2–5 min within the cell culture incubator.
3. Carefully aspirate Versene solution from the plate when the colonies are dissociated *into single cells* but still lightly attached to the flask.
4. Use 5 mL of StemMACS Brew medium with ROCK inhibitor at 5 μM to wash the colonies off the flask and count cell numbers using a hemocytometer.
5. Replate the cells on Matrigel-precoated flasks (optional VTN-N or LN521) at a density of 1 x 10^4^ cells/cm^2^, which corresponds to 2.5 × 10^5^ cells in a T25 flask (for all coating materials).

***Note:*** The duration of Versene-facilitated dissociation is dependent on cell confluency and should be closely examined under the microscope. The optimal time point to remove Versene solution is when the colonies are dissociated into single cells but still attached to the flask. If the cells are overtreated by Versene and the colonies are detached from the plate, add 2× volume of StemMACS Brew medium to dilute and centrifuge the cells at 200 x *g* for 3 min at 20°C. Aspirate the supernatant before resuspending and seeding the cells with StemMACS Brew medium plus ROCK inhibitor at 5 μM.

### Preparation of hiPSC differentiation

**Timing: 4 days, 10 min/day**

This step expands and conditions plated hiPSCs for subsequent differentiation.

6. Remove ROCK inhibitor containing medium and change to StemMACS Brew medium without ROCK inhibitor 2 days after passaging (day −2).
7. Again, refresh StemMACS Brew medium the next day (day −1).

**CRITICAL:** Cells should have reached a confluency of 100 % until day 0 to start differentiation. If not the case, adapt seeding numbers in a range between 0.5 × 10^4^ and 2 × 10^4^ cells/cm^2^ depending on proliferation rate. The critical parameter is reaching ∼90%–100 % confluency within 4 days. Confluency can be recognized by the formation of a uniform monolayer without visible gaps between cells, but before multilayering or detachment occurs.

### Mesoderm induction and differentiation of epicardial-like cells

**Timing: 10 days, 10–30 min/day**

This step induces epicardial-like intermediates that resemble developmental precursors of vSMCs and provide the foundation for downstream vSMC differentiation.

8. Day 0: After achieving a desired confluency of 100 %, wash the cells twice with pure RPMI 1640 plus GlutaMax medium. Replace the medium with mesoderm induction medium (MIM), using 10 mL per T25 flask.
9. Day 1: Refresh the medium on day 1.
10. Day 2: After culturing the cells in MIM for 48 h, wash them twice with pure RPMI 1640 plus GlutaMax medium. Then, replace the medium with 10 mL of mesoderm specification medium (MSM) per T25 flask.
11. Day 3 Replate:
  a. Wash cells once with TrypLE express (2 mL/T25 flask), then add prewarmed TrypLE express (2 mL/T25 flask) and incubate at 37°C for 3–5 min.
  b. Stop reaction by adding Epicardial specification medium (ESM), 5 mL/T25 flask. Dissociate cells by pipetting up and down *carefully.*
  c. Pool cells and centrifuge at 300 x *g* for 5 min. Aspirate supernatant and resuspend cell pellet in ESM.
  d. Count cells and plate them at 40.000 cells/cm^2^ in ESM (10 mL/T25) on Laminin-coated plates. Laminin should be used from day 3 regardless of the initial coating material.
12. Day 5, 7, 9: Refresh ESM every other day until day 10.

### Differentiation of epicardium-derived vSMCs

**Timing: 12 days, 10–30 min/day**

This step directs epicardial-like intermediates toward vSMC specification and promotes acquisition of a contractile phenotype suitable for downstream functional and molecular analyses.

13. Day 10: Change the medium to Smooth muscle medium (SMM), after washing the cells twice with KnockOut Basal medium (KBM).
14. Day 11, 12, 13: Replace SMM every day until day 14.
15. Day 14 Replate:
  a. Wash cells once with TrypLE express (2 mL/T25 flask), then add prewarmed TrypLE express (2 mL/T25 flask) and incubate at 37°C for 3–5 min.
  b. Stop reaction by adding SMM, 5 mL/T25 flask.
  c. Dissociate cells by pipetting up and down *carefully.*
  d. Pool cells and centrifuge at 300 x *g* for 5 min. Aspirate supernatant and resuspend cell pellet in SMM.
  e. Count cells and plate them at 80.000 cells/cm^2^ in SMM (10 mL/T25) on Vitronectin-coated plates. Vitronectin should be used from day 14 regardless of the initial coating material.
16. Day 16, 18, 20: Change medium every other day until day 22.

**CRITICAL:** Increased cell death is expected for the first 3 days of differentiation. Make sure to wash away all cell debris properly when exchanging medium.

**CRITICAL:** Laminin and vitronectin coating solutions dry out quickly. When aspirating medium, refill the flask immediately to prevent cell detachment.

***Note:*** TrypLE treatment duration is critical for a successful passaging step. If you keep it too short, cells will not detach properly and harsh pipetting will be needed to resuspend them, which means shear stress and damage to the cells. On the other hand, prolonged TrypLE treatment leads to apoptosis. The optimal time point to neutralize TrypLE is when the cells are dissociated into single cells but still lightly attached to the flask, which should be evaluated under the microscope thoroughly. Cells should easily be washed off the flask’s surface when rinsing with medium.

***Note:*** Differentiated vSMCs can be kept in culture with weekly passaging steps. Be aware that vSMCs have a limited proliferative capacity and will eventually stop dividing. We cultured vSMCs for up to 3 passages.

### Characterization of hiPSC-derived vSMCs

**Figure 3:**
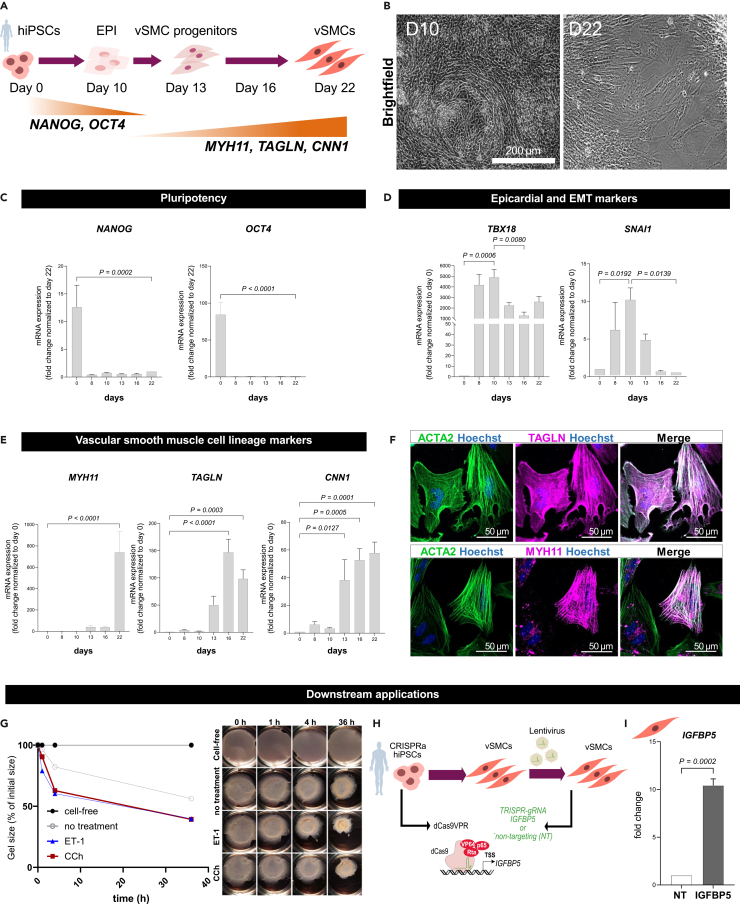
Characterization of hiPSC-derived vSMCs and their downstream application for functional validation (A) Schematic illustration depicting the differentiation of 2D hiPSC-derived vSMCs from hiPSCs. (B) Phase contrast microscopy showing the morphology of developing vSMCs at indicated time points D10 and D22 of differentiation. (C) Quantitative RT-PCR analysis of pluripotency markers (*OCT4, NANOG*) during the course of differentiation; n = 6 independent differentiations, results are normalized to TBP as housekeeping gene and expressed as fold change relative to D22, depicted as bar graphs with SEM. Statistical analysis was performed using one-way ANOVA. (D) Quantitative RT-PCR analysis of epicardial/EMT markers (*TBX18*, *SNAI1*) and (E) vSMC differentiation markers (*MYH11, TAGLN, CNN1*) during the course of differentiation; n = 3–6 independent differentiations, results are normalized to TBP as housekeeping gene and expressed as fold change relative to D0, depicted as bar graphs with SEM. Statistical analysis was performed using one-way ANOVA. (F) Immunofluorescence staining for vSMC markers ACTA2, MYH11 and TAGLN in D22 hiPSC-derived vSMCs. (G) Collagen contraction assay with response to vasoactive reagents such as Carbachol (CCh) at 1 mM and Endothelin 1 (ET-1) at 100 nM along with representative brightfield pictures of the wells. (H) Schematic illustration of the CRISPRa-mediated induction of *IGFBP5* expression. CRISPRa2.0-hiPSCs were differentiated to vSMCs and subsequently transduced with lentiviral particles containing *IGFBP5* or NT gRNAs, which guide the dCas9VPR complex to the transcription start site of *IGFBP5* to activate gene expression. (I) Quantitative RT-PCR *IGFBP5* expression analysis in IGFBP5-transduced vSMCs compared and normalized to respective NT control, 7 days post transduction; n = 3 independent transductions, results are normalized to TBP as housekeeping gene, depicted as mean with SEM. Statistical analysis was performed using an unpaired Student’s *t* test.

Fully differentiated hiPSC-vSMCs (Figures 3A and 3B) can be used for downstream analysis as immunofluorescence staining, transcriptional analysis as well as functional assays from day 22 onwards. We examined the loss of pluripotency markers (e.g., *NANOG, OCT4*) and the acquisition of vSMC lineage markers (e.g., *MYH11, TAGLN, CNN1*) using quantitative RT-PCR. Additionally, we verified the protein expression of vSMC markers ACTA2, MYH11, and TAGLN (Figures 3C–3F).

### Validated downstream applications

#### Collagen contraction assay for functional validation

17. Prepare a master mix by combining 2x DMEM and collagen I in a 1:1 v/v ratio.
18. Adjust the pH to neutral using sodium hydroxide (0.1 mg/mL), using phenol red as a pH indicator.
19. Dissociate 5 × 10^5^ cells as previously described, and add them to the master mix to achieve a final collagen concentration of 1 mg/mL.
20. Pipette 500 μL of the mixture into each well of a 24-well plate and incubate at 37°C for 45 min to allow hydrogel consolidation.
21. Carefully add 1 mL of SMSM to each well after incubation.
22. After 24 h, detach the vSMC patches from the well surfaces using a 10 μL pipette tip.
23. Treat the patches with either 1 mM carbachol (CCh) or 100 nM endothelin-1 (ET-1). Use untreated patches and cell-free collagen patches as controls. Capture images at 0, 1, 4 and 36 h (Figure 3G).

#### Target validation by CRISPRa-mediated transcriptional activation in hiPSC-derived vSMCs

CRISPR/dCas9 fused to the VPR activator enables potent activation of endogenous genes by precisely targeting genomic loci for perturbation and validation studies. Using the above-described protocol allowed to differentiate previously described CRISPRa-hiPSCs into vSMCs, and gene activation was carried out according to our previously established protocol for CRISPRa2.0 hiPSC-derived cardiomyocytes.^5^^,^^6^^,^^7^ For validation, insulin-like growth factor binding protein 5 (*IGFBP5*) was selected. The objective was to demonstrate gene activation and validate endogenous gene expression characteristics of hiPSC-derived vSMCs.^8^

Briefly described, oligonucleotides encoding either the *IGFBP5* 5′ TSS-targeting guide (g)RNA or a non-targeting (NT) control (sequences provided in resources section) were cloned into the TRISPR vector and used for lentiviral production, following the method described in detail before.^6^ hiPSC-CRISPRa2.0-derived vSMCs were transduced with the lentiviral particles containing either the *IGFBP5*-gRNA or the NT control. After 7 days, cells were collected for RNA extraction and transcript analysis by quantitative RT-PCR (Figures 3H and 3I). All experiments were conducted in triplicates, and gene activation was quantified as the fold increase relative to CRISPRa2.0-vSMCs transduced with NT gRNAs. This demonstrates that the differentiated CRISPRa-vSMCs retained functional responsiveness to targeted gene activation, supporting the reliability of the vSMC differentiation protocol.

## Expected outcomes

After an initial increase in cell death during the first three days of differentiation, the cells begin to show an increased proliferation rate and improved survival from the first passaging onwards. Around day 6, as the colonies grow larger, crescent-shaped formations start to appear, eventually giving rise to cobble-stone-like epicardial cells. By day 10, extensive cell aggregates begin to overgrow the underlying cell layer. After the second passaging on day 14, the cells have adopted a vSMC phenotype, characterized by elongated and spindle-shaped morphology. At this stage, the cells are still proliferating and fully differentiated vSMCs can be obtained by day 22 for downstream analysis.

The purity of vSMCs can vary among different hiPSC lines and across different batches of differentiation. However, most experiments following this protocol can achieve greater than 80 % purity. Purity can be assessed by evaluating cell morphology under a phase contrast microscope and quantified through vSMC marker immunofluorescent staining, imaging, or functional assays that measure contraction capacity.

The cell count for a 25 cm^2^ flask on day 22 of differentiation can vary, typically ranging from 4 to 8 million cells. The total RNA yield usually ranges between 15 and 30 μg.

Cells can be maintained in culture for up to three passages with weekly splitting procedures. However, the proliferation capacity of vSMCs is limited and tends to decrease over time. If the cells exhibit unusually high proliferation rates, it may be necessary to reevaluate marker expression as contamination with fibroblast-like cells could be a possibility.

## Limitations

This vSMC differentiation protocol has been successfully reproduced over several batches of hiPSCs (LiPSC-GR1.1, TC-1133). Our serum-free differentiation media offer a significant advantage, providing a chemically defined environment with elimination of batch-to-batch variability typically associated with use of serum. Due to differences in proliferation rates, seeding numbers should be optimized for each cell line.

One major challenge is the phenotypic plasticity of vSMCs, which can exhibit a wide range of phenotypes depending on environmental cues, making it difficult to achieve a stable, terminally differentiated state *in vitro*. The immaturity of these cells in contrast to primary adult cells further complicate the reproducibility and translational potential of these models. Also, environmental factors such as culture media composition and especially mechanical influences can affect the expression of key vSMC markers, leading to phenotype switching.

With occurrence of heterogenous differentiations with a relevant portion of fibroblast-like cells vSMC-differentiations can be overgrown by these due to higher proliferation rates. As there is no selection protocol to remove fibroblast remains from culture, this can only be overcome by for example MACS sorting with vSMC-specific antibodies.

## Troubleshooting

### Problem 1

hiPSCs are not overgrowing the whole flask, they leave blank spaces.

### Potential solution

Sufficient plate coating is essential for hiPSCs to attach properly and survive. Ensure that the whole flask’s surface is covered with the corresponding coating solution and do not let it dry out during preparation process (see also: plate coating for hiPSC maintenance and mesoderm differentiation).

### Problem 2

hiPSCs have not reached a desired confluency of 90% until day 0 for start of differentiation.

### Potential solution

hiPSC-confluency at differentiation starting point is critical for the successful derivation of vSMCs. If cultured hiPSCs did not reach the desired confluency of about 90% until that point, waiting for one more day is an option. If hiPSCs are still not confluent enough, we recommend to re-passage the cells and adapt seeding density. Do not start mesoderm induction at low density levels (see also: maintenance and passaging of hiPSCs).

### Problem 3

Many cells die during the differentiation process.

### Potential solution

Increased cell death is an expected phenomenon throughout the first days of differentiation. Underlying causes are:

• After differentiation start at already high confluency cells are still proliferating, leading to over-confluency and consequential cell death.
• IWP-4 as a potent Wnt signaling inhibitor is added during mesoderm induction to induce a cardiac fate. Cells turning spontaneously into other lineages still in need for active Wnt signaling undergo apoptosis.

Thoroughly wash away all dead cells when changing medium (see also: mesoderm induction and differentiation of epicardial-like cells). Cell death should stop after first passaging on day 3 of differentiation. In case of persistence, consider a bacterial contamination or insufficient coating as potential reasons. Re-start differentiation in these cases.

### Problem 4

vSMCs stop proliferating and cannot be passaged again.

### Potential solution

While vSMC progenitors do show a sufficient proliferation rate, a reduced proliferation rate is typical for differentiated vSMCs. At some point they will stop proliferating, however, the exact time point is variable. We kept vSMCs in culture for up to 3 passages, but this is not necessarily the case for every differentiation.

### Problem 5

Cells in differentiation detach as whole cell layers from the flasks surface.

### Potential solution

This can be observed especially after seeding cells on Laminin or Vitronectin layers. These solutions dry out *very* quickly. While changing medium, make sure to always refill flasks directly before emptying others. This way a dry out can be prevented effectively (see also: alternative coating for hiPSC maintenance and coating instructions for epicardial and vSMC derivatives).

Detachment can also be observed when differentiations are proliferating fast, leading to high cell densities. vSMC-autonomous contractile forces are then leading to a contraction of the whole cell sheet with consequential detachment. Consider earlier passaging in these cases.

## Resource availability

### Lead contact

Further information and requests for resources and reagents should be directed to and will be fulfilled by the lead contact, Laura Cecilia Zelarayán (laura.zelarayan@med.uni-goettingen.de).

### Technical contact

Further technical information and requests should be directed to and will be fulfilled by the technical contact, Laura Priesmeier (laura.priesmeier@med.uni-goettingen.de).

### Materials availability

This study did not generate new unique reagents.

### Data and code availability

The published article includes all datasets generated or analyzed during this study.

## Acknowledgments

This work was supported by the 10.13039/501100001659DFG grants CRC1213 (B10N) and ZE 900/7-1 and the 10.13039/100010447German Centre for Cardiovascular Research e.V. (DZHK).

## Author contributions

L.P.: writing – original draft, visualization, validation, methodology, and investigation; M.T.: review, editing and resources; L.C.Z.: writing – review and editing, supervision, funding acquisition, and conceptualization.

## Declaration of interests

The authors declare no competing interests.

## References

[1] Villa-Diaz L.G., Ross A.M., Lahann J., Krebsbach P.H. (2013). Concise review: The evolution of human pluripotent stem cell culture: from feeder cells to synthetic coatings. Stem Cell..

[2] Chen G., Gulbranson D.R., Hou Z., Bolin J.M., Ruotti V., Probasco M.D., Smuga-Otto K., Howden S.E., Diol N.R., Propson N.E. (2011). Chemically defined conditions for human iPSC derivation and culture. Nat. Methods.

[3] Melkoumian Z., Weber J.L., Weber D.M., Fadeev A.G., Zhou Y., Dolley-Sonneville P., Yang J., Qiu L., Priest C.A., Shogbon C. (2010). Synthetic peptide-acrylate surfaces for long-term self-renewal and cardiomyocyte differentiation of human embryonic stem cells. Nat. Biotechnol..

[4] Preissner K.T. (1991). Structure and biological role of vitronectin. Annu. Rev. Cell Biol..

[5] Priesmeier L., Schoger E., Cyganek L., Cecilia Zelarayán L. (2023). Combining a tetracycline (Tet)-inducible gRNA system and CRISPRa for titratable and timely controlled enhancement of endogenous SHISA3 activation in human induced pluripotent stem cells (hiPSC). Stem Cell Res..

[6] Schoger E., Argyriou L., Zimmermann W.-H., Cyganek L., Zelarayán L.C. (2020). Generation of homozygous CRISPRa human pluripotent stem cell (hiPSC) lines for sustained endogenous gene activation. Stem Cell Res..

[7] Schoger E., Zimmermann W.H., Cyganek L., Zelarayán L.C. (2021). Establishment of a second generation homozygous CRISPRa human induced pluripotent stem cell (hiPSC) line for enhanced levels of endogenous gene activation. Stem Cell Res..

[8] Xu Q., Li S., Zhao Y., Maures T.J., Yin P., Duan C. (2004). Evidence that IGF binding protein-5 functions as a ligand-independent transcriptional regulator in vascular smooth muscle cells. Circ. Res..

